# Optimizing operation of delivering and fetching wagons at a railway station with mixed-shaped goods operation sites

**DOI:** 10.1371/journal.pone.0263029

**Published:** 2022-01-31

**Authors:** Chuijiang Guo, Shengdong Li

**Affiliations:** School of Logistics, Chengdu University of Information Technology, Chengdu, China; Southwest Jiaotong University, CHINA

## Abstract

The problem of delivering and fetching wagons at a railway station with mixed-shaped goods operation sites is considered, with a view to minimizing the running time between goods operation sites and waiting time of the locomotive in the planning period as the optimization objective. A general mathematical model for delivering and fetching wagons at a railway station with mixed-shaped goods operation sites has been formulated. The methods of judging and processing reverse of delivering and fetching wagons, dividing batches, and judging number of wagons for batch operation are provided to determine the feasibility of the solution, and an improved simulated annealing algorithm is introduced as our algorithm to the model. Finally, an experimental station is taken as an example to verify the model and algorithm. The results show that simulated annealing algorithm is relatively superior in computational efficiency and result compared with genetic algorithm and tabu search algorithm, the computing time of the algorithm provided can meet the requirements of planning shunting operations in railway station, and the model proposed is universal for other layout forms of GOSs and different operation forms.

## Introduction

**Fig 1:**
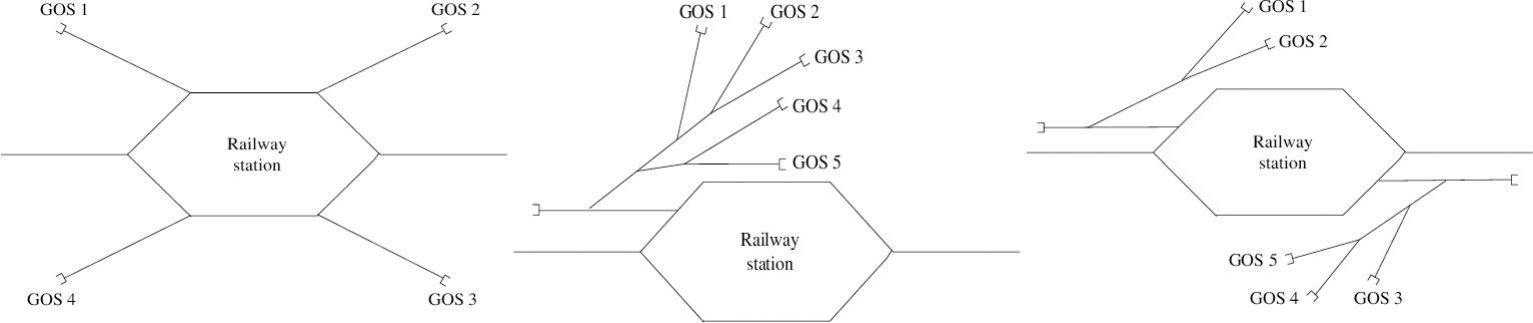
Layout of GOS in railway stations. (a) Radial-shaped Station, (b) Branch-shaped Station, (c) Mixed-shaped Station.

Most of China’s railway stations need to handle freight business, so they are equipped with freight yards, special lines and other goods operation sites(hereinafter collectively referred to as GOSs). The shunting locomotive needs to deliver and fetch wagons between the railway station and GOSs. Whether a technical station or an intermediate station, the railway station can be divided into three types according to the layout of the GOSs: radial-shaped, branch-shaped and mixed-shaped, as shown in Fig 1.

In the 1950s, Professor Ma Xu put forward the concept of delivering and fetching wagons in written form. Since then, scholars have gradually deepened the research on the issue. Scholars have done a lot of research on formulating the operation plan of delivering and fetching wagons in railway stations with radial-shaped GOSs. For example, scheduling theory [1] and the system description method [2] are used to solve it with the wagons of a direct traffic flow. There are also studies on the non-direct traffic flow with the optimization objective of minimizing the total dwell time of wagons in the station [3]. Jaehn, F.et al. studied the shunting operation planning of a train from the wagon park to the maintenance plant, which is similar to the problem studied in this paper [4].

At present, the existing methods for the problem of delivering and fetching wagons in a railway station with branch-shaped GOSs are transforming it into the shortest path problem of a Hamiltonian graph [5] or scheduling theory [6], and most of them are based on the former method [7–10].The optimization objective is widely claimed to be minimizing the dwell-time of wagons at the station or the running distance of the shunting locomotive [11].Some scholars take the sequence and batch division of wagons as a system to optimize [12–14], which promotes the process of studying the theory of formulating the shunting operation plan combined with the actual work.

In some developed countries, the research on optimizing delivering and fetching wagons is mainly concentrated on the field of road transportation due to the relatively developed road and air transportation, and there is little research on delivering and fetching wagons for railway transportation. Because the sequence optimization of delivering and fetching wagons among GOSs is a vehicle routing problem, some findings of road transportation could provide some valuable ideas for the research on the issue. Mitrovic-Minic formulated a multi-objective optimization model for the large-scale dynamic pickup and delivery problem with time window constraints, and the corresponding heuristic algorithm is proposed to solve it [15]. Zachariadis studied the vehicle routing problem (LDVRP) related to vehicle load. When the scale of LDVRP is small, it is solved by branch-and-cut method. When the scale of the problem is large, the local search algorithm is used to solve it [16]. Sartori and Buriol proposed an improved hybrid algorithm based on mathematical programming, and a new method to generate instances for routing problems based on open data [17].

In this paper, for the problem of delivering and fetching wagons at railway stations with mixed-shaped goods operation sites, with the aim of minimizing the running time and waiting time of locomotives in the planned period as the optimization objective, and considering i) the conflicts between arriving or departing train routes and shunting routes, ii) the visiting sequence required for transferring wagons between GOSs, iii) the operating sequence required by the same wagons in a certain GOS to be delivered first and then fetched, iv) the traction capacity of the shunting locomotive, v) the capability of the GOS, vi) the time when wagons are separated from the train, and vi) the time of the wagons returning to the station coupled with their departing train, a general mathematical model for the delivering and fetching of wagons at railway stations with mixed-shaped goods operation sites is formulated. We resolved it with an improved simulated annealing algorithm. At present, there is no general model of the shunting operation plan for a railway station with mixed-shaped GOSs which includes route constraints and could be compatible with the situations of wagons arriving and departing simultaneously and dispersedly, different operation types, and all forms of operation. As far as I know, the model of optimizing delivering and fetching wagons at railway stations with mixed-shaped GOSs is firstly put forward, and the conflicts between arriving or departing train routes and shunting routes is firstly considered in the models of optimizing delivering and fetching wagons in the GOSs. Therefore, it will deepen the research on the problem of delivering and fetching wagons at railway station.

This paper is structured as follows. The analysis on the process of delivering and fetching of wagons in a railway station with mixed-shape GOSs is given in Section 2. Our optimization model is presented in Section 3. The algorithm we provide is described in Section 4. In Section 5, we take example of an experimental station and compare with other two algorithms to discuss the superiority of the algorithm, and we come to conclusions in Section 6.

## Analysis on the process of delivering or fetching wagons

The problems studied in this paper can be described as: In a railway station with mixed-shaped GOSs, for a certain period, there are several waiting wagon groups with the same or different arrival and disassembling time, and there are several waiting groups with the same or different loading or unloading time and marshalling time at some GOSs, and there are maybe some dual operation wagon group need to be transferred for some certain GOSs. It is required to prepare a reasonable plan for delivering and fetching wagons operation batches, delivering and fetching time and sequence scheme in each batch, to ensure that the total cost of delivering and fetching wagons in the period is minimized on the premise of meeting the constraints such as shunting traction capacity, capacity of the GOS and wagon flow connection requirements, and so on.

For a shunting operation at a station with a radial-shaped GOS, after the task of delivering or fetching a wagon for the GOS has been completed, the shunting locomotive must go back to the railway station before another GOS can be serviced. Therefore the entry times of wagons at each GOS are different, as are the return times to the railway station. In a railway station with a branch-shaped GOS, after the shunting locomotive has finished the task at the GOS, it does not need to go back to the railway station before servicing another, and only returns to the station after all tasks have been completed. The arrival times of wagon groups at each GOS are therefore different, but the return times to the station are the same. According to statistics, railway stations with mixed-shaped GOSs account for the largest proportion of ordinary-speed railway stations in China. Mixed-shaped GOSs can be regarded as a combination of radial-shaped and branch-shaped GOSs, and have characteristics of both of them. In other words, a radial-shaped or branch-shaped GOS can be considered as a special form of mixed-shaped GOS. If there is only one GOS in each branch, a railway station with a mixed-shaped GOS can be regarded as having a radial-shaped GOS. If there is only one branch in the station and the number of GOSs of the branch is more than two, then a railway station with a mixed-shaped GOS can be regarded as having a branch-shaped GOS. Therefore, the research on delivering and fetching wagons in a railway station with a mixed-shaped GOS is also applicable for a railway station with radial-shaped and branch-shaped GOSs.

The vast majority of GOSs in railway stations connect with the railway station from the throats at both ends of the station. The distance between tracks is relatively much shorter than the arrival‒departure tracks(or shunting tracks).For a railway station with mixed-shaped GOS, all GOSs are distributed in a radial shape on the whole, but for GOSs within a certain branch, they are distributed in a branch shape.

If the station is equipped with two or more GOSs, these may be in the same quadrant of the station or in different quadrants. However, if they are on the same side of the main track of the station, there may be no route interference in the shunting operations conducted for the arriving and departing trains, on condition that the layout of the railway station is reasonable.

However, if the GOSs are located on both sides of the main track, it is inevitable that the locomotive will cross the main track during the operation of cross-branch shunting. This may interfere with the operation of receiving and departing trains, and the operation routes between the shunting operations and receiving and departing trains must be staggered in time and in space.

## Model formulation

### Scenario setting

Because there are various operation scenarios in actual work, the problem of delivering and fetching wagons will be only discussed in this paper under the following conditions.

1. Wagons that are disassembled at the same time and sent to a certain GOS in the same batch, or wagons that are taken out from the same GOS and grouped to the same departure train, should be regarded as a certain wagon group. Any wagon group could not be split in the whole plan period.
2. The time when the trains to which each wagon belongs are disassembled, the time when the loading and unloading operations are completed, and the start time of the marshalling operation of the corresponding trains that each wagon group is coupled to are known in advance.
3. The number of the wagons to be delivered and fetched at each GOS in the plan period are known in advance.
4. The accommodation capacity of each GOS and the traction capacity of the shunting locomotive are known in advance.
5. The time required for selecting a wagon group, the time for adjusting a wagon group to its goods position, the time spent by the locomotive running among GOSs(or railway stations), and the times spent by each wagon group on loading or unloading are known in advance.
6. After the shunting locomotive has sent the wagon group to its GOS, it will leave immediately. There is no need to wait for the loading and unloading of the wagon before leaving the GOS.
7. The shunting locomotive visits the same GOS only once in a batch of operations.
8. Only one shunting locomotive is available at the railway station for delivering and fetching wagons.

### Symbol definition

In order to formulate and solve the model of the delivering and fetching of wagons at a railway station with mixed GOSs, the following symbols are introduced to represent the parameters and variables.

1. **Symbols of parameters**
  *v*_0_:Railway station or shunting yard.
  *v*_*i*_, *v*_*i*’_:GOSs.
  *I*:Number of GOSs.
  *V*_*k*_: GOS branch, which represents the set of corresponding GOSs.
  *K*:Number of GOS branches.
  *t*_*ii*’_:Measured time for the locomotive running between the GOSs (station).
  *t*^*traverse*^:Time for the shunting locomotive traversing the main track.
  $\left[t_{w},t_{w}^{'}\right]$:Shunting time-window.
  *T*_*s*_:Start time of the planning period.
  *T*_*e*_:End time of the planning period.
  $t_{i}^{arr}$:Time when the wagon group that should be loaded or unloaded at GOS *v*_*i*_ has been disassembled.
  $t_{i}^{finish}$:Time when the wagons of the GOS *v*_*i*_ have been loaded or unloaded.
  $t_{i}^{mar}$:Start time of the marshalling operation for the trains to which the wagons of GOS *v*_*i*_ should be coupled.
  $t_{t}^{j}$:The time required to selecting wagon group at the beginning of batch *j*.
  $t_{f}^{j}$:The time of batch *j* required to split wagon group after shunting locomotive return the station.
  $t_{s}^{j}$:The time of batch *j* required to collect wagons before fetching wagons(including fetching wagons of transferring operation).
  $t_{d}^{j}$:The time of batch *j* required to adjust position of wagons after delivering wagons(including delivering wagons of transferring operation).
  *W*:Number of shunting time-windows.
  *q*_*i*_: Number of wagons that should be fetched for GOS *v*_*i*_.
  *p*_*i*_: Number of wagons that should be delivered for GOS *v*_*i*_.
  *Q*:Maximum traction capacity of the shunting locomotive.
  *R*_*i*_:Capacity of GOS *v*_*i*_.
  $n_{k}^{j0}(i)$:Number of wagons remaining in GOS *v*_*i*_ of branch *k* before the batch *j*.
  *l*:Work mode of delivering and fetching wagons. If the task is delivering the wagons, *l* is equal to 1.If the task is fetching the wagons that have reached the railway station during the planning period, *l* is equal to 2. If the task is fetching the wagons that remain at the GOS at the beginning of the planning period, *l* is equal to 3.
  $g_{k}^{l}(i)$:Operation with mode *l* of the shunting operation required for the locomotive at GOS *v*_*i*_ of the branch *k*.
  $b[g_{k}^{l}(i)]$:Wagon group corresponding to the operation of delivering and fetching wagons $g_{k}^{l}(i)$.
  *U*:Total number of shunting operations in the whole planning period.
2. **Symbols of variables**
  $t_{j}^{start}$:Start time of the batch *j*.
  $t_{i}^{arrive}$:Time when the shunting locomotive reaches GOS $v_{i}$.
  $t_{i}^{leave}$:Time when the shunting locomotive leaves GOS *v*_*i*_.
  $t_{j}^{return}$:Time when all the wagon groups of batch *j* return to the railway station.
  $x_{ii'}^{j}$:Binary variable such that if the locomotive runs from GOS *v*_*i*_ (or station) to GOS *v*_*i*’_ (or station) during the operation of batch *j*,then $x_{ii'}^{j}$ is equal to 1,otherwise 0.
  *y*_0*i*_:Binary variable, which respectively indicates whether the shunting locomotive should cross the main track when going from the station *v*_0_ to the GOS *v*_*i*_ or go back to the station *v*_0_ from the GOS *v*_*i*_.If it is essential to for the locomotive to cross the main track, *y*_0*i*_ = 1 or *y*_*i*0_ = 1, otherwise, *y*_0*i*_ = 0 or *y*_*i*0_ = 0.
  *S*:A solution which represents serial of the operations of delivering and fetching wagon according to the operation sequence.
  $d[g_{k}^{l}(i)]$:Serial number of shunting operation $g_{k}^{l}(i)$ in the solution.
  *J*:Number of batches.
  *V*_*j*_:Set of GOSs of batch *j*.

### Constraints

(1) Visiting sequence constraint of locomotive required by the transferring operation

In order to reduce the deadhead kilometers of wagons, improve the utilization efficiency of wagons, and reduce the average dwell-time of wagons at the railway station per goods operation, the empty wagons generated after unloading should be used to load goods as much as possible. Therefore, a certain number of wagons for dual goods operation need to be transferred from the site for unloading goods to the site for loading goods. Only by visiting the unloading GOS to take out the empty wagons can the corresponding loading GOS make use of these wagons. In other words, a priority visiting sequence constraint exists in the operation of delivering and fetching wagons, which can be expressed as Eq (1).


$$d[g_{k}^{3}(i)]<d[g_{k'}^{1}(i')]\;if\;b[g_{k}^{3}(i)]=b[g_{k'}^{1}(i')]$$ (1)

(2) Constraints for the same wagon group of a GOS being delivered first and then fetched

Any wagon group in the same GOS should meet the sequence requirement that delivering a wagon should be executed first before fetching. In other words, delivering wagons, loading or unloading goods, and fetching wagons should be executed sequentially in the delivering and fetching wagon operation. Therefore, fetching a wagon must be carried out after delivering the wagon, and these canbe represented as Eq (2).


$$d[g_{k}^{1}(i)]<d[g_{k}^{2}(i)]\;if\;b[g_{k}^{1}(i)]=b[g_{k}^{2}(i)]$$ (2)

(3) Constraints for traction capacity of shunting locomotive

The number of wagons connected with the locomotive for each shunting operation batch cannot exceed the traction capacity of the locomotive, otherwise it will cause difficulties with starting and braking of the locomotive. The traction ability of the locomotive will affect the batch division of the delivering and fetching of wagons. For the same task, the shunting operation may need to be divided into more batches for a locomotive with smaller power, which can be expressed as Eq (3).


$$\sum_{v_{i}\in V_{j}}p_{i}+\sum_{h=1}^{i}\left(p_{i}-q_{i}\right)\leq Q\;i=1,2,\ldots ,I\;j=1,2,\ldots ,J$$ (3)

(4) Constraints for capacity of GOS

Due to the limited capacity of the goods space, the number of wagons available at any time cannot exceed the capacity of the GOS. The capacity of the GOS will also affect the batch division of the delivering and fetching wagon operation, which can be expressed as Eq (4).


$$n_{k}^{j0}(i)+p_{i}-q_{i}\leq R_{i}\;i=1,2,\ldots ,I\;j=1,2,\ldots ,J$$ (4)

(5) Constraints for the end time of batch operation

After the wagons are retrieved from the station, they should be coupled with the corresponding departing train. Therefore, the time for the wagons returning to the station must not be later than the latest marshalling time of the wagons, otherwise the departing train will be delayed or the wagons’ dwell-time at the station will be extended because they will then need to be coupled with an unplanned train, and this can be expressed as Eq (5).


$$t_{j}^{return}\leq \min\{t_{i}^{mar}\}\;v_{i}\in V_{j}$$ (5)

(6) Constraint for start time of a batch of operation

1) A wagon sent to the GOS in each batch operation can only be selected from the wagons that have arrived at the station and disassembled before the delivering of wagons; that is, the delivering and fetching operation of each batch operation which contains the operation of delivering wagons must start no earlier than the disassembly time of all the wagon groups that are to be delivered, and this can be expressed as Eq (6).


$$t_{j}^{start}\geq t_{i}^{arr}\;v_{i}\in V_{j}$$ (6)

2) The start time of each batch of wagons for delivering and fetching should not be earlier than the time at which the locomotive has returned to the marshalling yard (station)when the last batch of delivering and fetching of wagons is completed, and this can be expressed as Eq (7).


$$t_{j}^{start}\geq t_{j-1}^{return}\;j=1,2,\ldots ,J$$ (7)

3) The start time of the first batch of delivering and fetching of wagons should not be earlier than the start time of the planning period, which can be expressed as Eq (8).


$$t_{1}^{start}\geq T_{s}$$ (8)

4) If the first item of a batch operation requires the main track to be crossed, the start time of the batch must be within the shunting time-window, and the crossing time must be completed within the shunting time-window. This can be expressed as Eq (9), where *M* is a sufficiently large positive number.


$$M(y_{0i}-1)+t_{w}\leq t_{j}^{start}\leq t_{w}^{'}-t^{traverse}+M(1-y_{0i})\;v_{i}\in V_{j}$$ (9)

### Optimization objective

Based on the existing literature, it is proposed to take the minimum wagon-hours as the optimization objective. In fact, under the premise that the arrival and departure time of the train that the wagon group belongs to cannot be adjusted, the wagons’ total dwell-time in the planning period is determined. The duration of delivering and fetching the wagons of each batch refers to the period from the time the locomotive leaves the station (shunting yard) to the time it returns to the station (shunting yard) after the completion of the delivering and fetching of the wagons of a certain batch. The total time of delivering and fetching wagons is the sum of the time for delivering and fetching the wagons of all batches during the same planning period. The lower the total operation time of all the batches of the shunting operation, the higher the operation efficiency of the shunting, and the greater the time interval between batches for recuperation or other shunting activities during the planning period.

In this paper, the optimization objective is to minimize the total operation time for completing all batches of delivering and fetching wagons in the planning period. The total operation time of the shunting locomotive is composed of the running time of the shunting locomotive between GOSs (station or yard) and the time that related operations spent, the waiting time due to the uncompleted loading or unloading of goods when the shunting locomotive arrives at the GOS, and the interference time of the arriving and departing train in the shunting operation.

The first part of the optimization objective in this paper is to minimize the sum of the running time of the locomotive among GOSs (stations or shunting yards)for all batches, and the time spent by related operations, which concludes the time spent on selecting wagon group at the beginning of each batch $t_{t}^{j}$, the time spent on splitting wagon group after shunting locomotive return the station $t_{f}^{j}$, the time spent on collecting wagons before fetching wagons(including fetching wagons of transferring operation) $t_{s}^{j}$, and the time spent on adjusting position of wagons after delivering wagons(including delivering wagons of transferring operation) $t_{d}^{j}$. Those could be expressed as Eq (10).


$$f_{1}=\sum_{j=1}^{J}(\sum_{v_{i}\in \{V_{j},\;v_{0})}\sum_{v_{i'}\in \{V_{j},v_{0}\}}x_{ii'}^{j}t_{ii'}+t_{t}^{j}+t_{f}^{j}+t_{s}^{j}+t_{d}^{j})$$ (10)

The waiting time for loading and unloading the goods of the locomotive at the GOS refers to the non-production time between the time the locomotive arrives at the GOS and the time by which the wagons have been loaded and unloaded, the loading and unloading equipment has been evacuated and the condition for fetching wagons has been met. For a GOS, when the locomotive arrive at a GOS, the waiting time is $t_{i}^{finish}-t_{i}^{arrive}$ on condition that relevant goods operations have not been completed. Otherwise, the locomotive does not need to wait and the waiting time of it is zero. Therefore the second part of the objective of the optimization model in this paper is the sum of the waiting time for loading and unloading the goods from all batches, which can be expressed as Eq (11).


$$f_{2}=\sum_{j=1}^{J}\sum_{v_{i}\in V_{j}}\max(t_{i}^{finish}-t_{i}^{arrive},0)$$ (11)

If there is any conflict between the arriving or departing train and the shunting operation, it is necessary to stop all shunting operations that may affect the arriving or departing train at the station before preparing the routes of the arriving or departing train. Routes of shunting operations that may affect the arriving and departing train at a station include shunting routes that cross the main track, shunting routes that occupy the arrival and departure track, and shunting routes that do not occupy the arrival and departure track but still affect the arriving and departing train. For the convenience of the study, the shunting routes that may influence the arriving and departing train in this paper only refer to the those routes that cross the main track.

The segment unlocking route is not considered in this paper, that is, the routes of the arriving or departing train are handled once and unlocked once. If there is a space or time conflict between the shunting route and the route of the arriving and departing train, or the time remaining in the shunting time-window is insufficient for the locomotive to complete crossing the main track, the locomotive must halt in front of the corresponding protection signal and wait until the completion of the arriving or departing train.

During a batch of operations, the locomotive needs to travel from the station to the GOS, or return to the station from the GOS. If the locomotive goes between the GOSs of different branches, the locomotive also needs to return to the station from the first GOS and then run to another GOS. If the conflicting operation of an arriving and departing train is in progress when the locomotive is moving from the station to the GOS, this can be resolved by delaying the start time of the batch for the shunting operation. However, when the train goes back to the station *v*_0_, if the arriving and departing train is in conflict with the shunting operation that is in progress, the locomotive must wait for completion of the arriving and departing train operation in front of the corresponding protection signal. If the end time of the batch $y_{j}^{return}$ is not within the time interval $\left[t_{w},t_{w}^{'}-t^{traverse}\right]$, the length of time for which the arriving and departing train has influenced the shunting operation should be considered. The third part of the optimization objective in this paper is the interference time on the arriving and departing train of all batches by the shunting operation, which can be expressed as Eq (12).


$$f_{3}=\sum_{j=1}^{J}y_{i0}(t_{w}-t_{j}^{return})$$ (12)

The objective for the optimization model is the sum of delivering and fetching wagons time of all batches.A batch of delivering and fetching wagon operations refers to the technical operation process whereby the shunting locomotive returns to the station after fetching, delivering and transferring wagons from the relevant GOSs. The start time of batch *j* is $t_{j}^{start}$, the finish time of batch *j* is $t_{j}^{finish}$, and therefore, the duration of batch *j* is $t_{j}^{finish}-t_{j}^{start}$. In other words, it is the sum of the above three optimization objectives. the objective for the model can be expressed as Eq (13).


$$\min f(S)=\sum_{j=1}^{J}(t_{j}^{return}-t_{j}^{start})$$

$$=\sum_{j=1}^{J}\left(\sum_{v_{i}\in \{V_{j},\;v0\}}\sum_{v_{i'}\in \{V_{j},v_{0}\}}x_{ii'}^{j}t_{ii'}+t_{t}^{j}+t_{f}^{j}+t_{s}^{j}+t_{d}^{j}+\sum_{v_{i}\in V_{j}}\max(t_{i}^{finish}-t_{i}^{arrive},0)+y_{i0}(t_{w}-t_{j}^{return}\right))$$ (13)

Therefore, the optimization model of delivering and fetching wagons at a railway station with mixed GOSs can be listed as follows.


$$\min f=\sum_{j=1}^{J}\left(\sum_{v_{i}\in \{V_{j},\;v0\}}\sum_{v_{i'}\in \{V_{j},v_{0}\}}x_{ii'}^{j}t_{ii'}+t_{t}^{j}+t_{f}^{j}+t_{s}^{j}+t_{d}^{j}+\sum_{v_{i}\in V_{j}}\max(t_{i}^{finish}-t_{i}^{arrive},0)+y_{i0}(t_{w}-t_{j}^{return}\right))$$

S.t. Eqs (1)–(9)

In the above optimization model, $x_{ii'}^{j}$, $t_{j}^{start}$, $t_{i}^{arrive}$, $t_{i}^{leave}$, $t_{j}^{return}$ are decision variables, the other variables are intermediate variables.

## Algorithm to the model

The optimization model of delivering and fetching wagons at railway stations with mixed GOSs is a decision problem that needs to determine the batch division, operation sequence, and starting time of operation. In this paper, in order to search for satisfactory solutions,a simulated annealing algorithm will be used, and the constraints will be considered to judge whether the solution is feasible.

### Judging and processing reverse of delivering and fetching wagons

**Fig 2:**
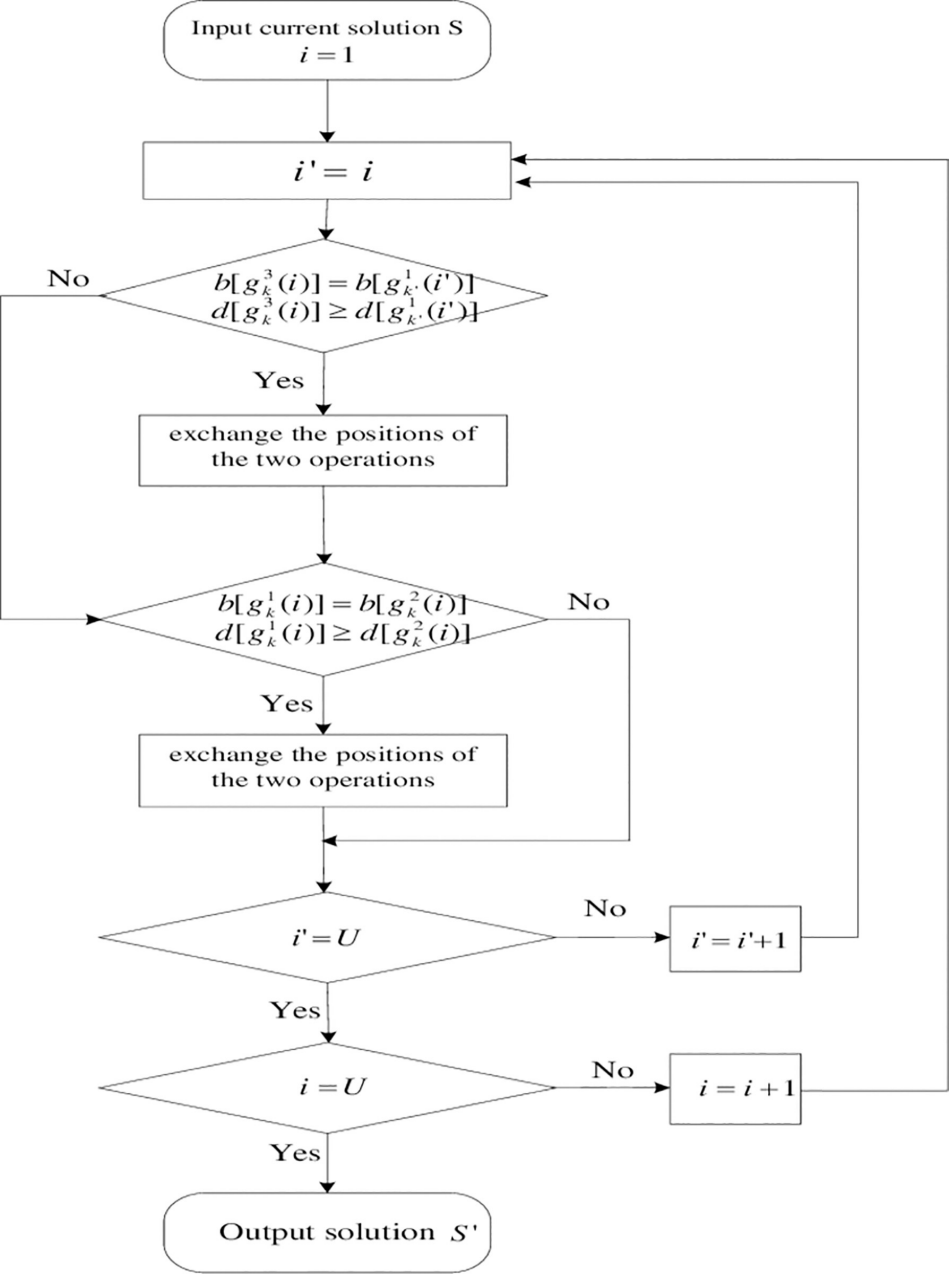
Flow chart of judging and processing reverse of delivering and fetching wagons.

If the wagon group of the latter operation of delivering wagons is the same as the current fetching wagon operation and their GOSs are the same, exchange the positions of the two operations in the solution. If the wagon group of the latter fetching wagon operation is the same as the current delivering wagon operation, but the GOSs are different, exchange the two operations in the solution. The process of the reverse judging and processing function of delivering and fetching of wagons are shown as Fig 2.

### Dividing batches

**Fig 3:**
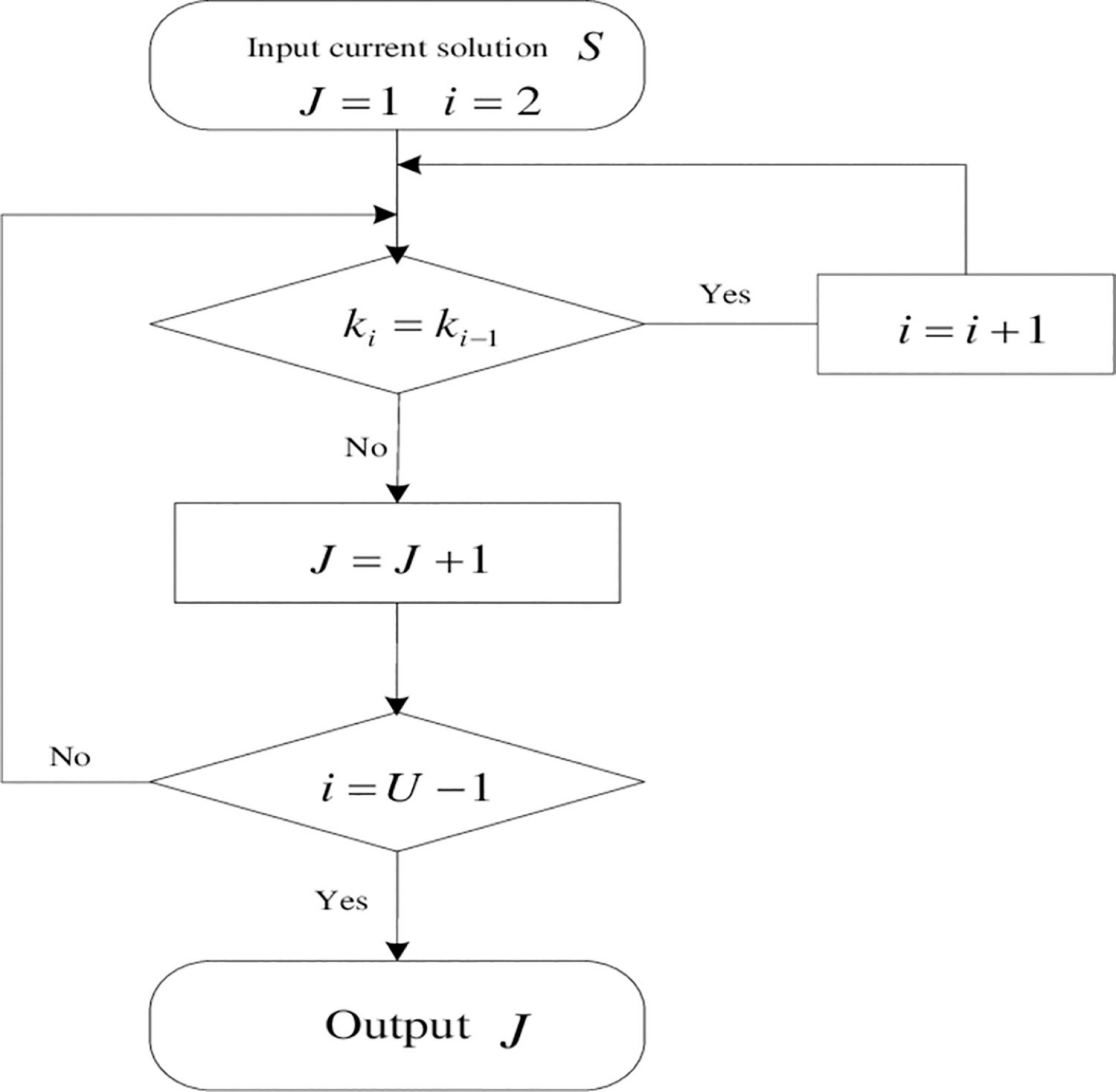
Flow chart of dividing batches.

During the shunting operation in the planning period, when the shunting locomotive returns to the station, the last batch of the shunting operation ends. When the locomotive starts from the station again, the next batch of shunting operation starts. Inspect the delivering and fetching wagon operations one by one until the last, and record the number of the batch of the current solution. If the branch of the current operation is the same as that of the previous operation, namely *k*_*u*_ = *k*_*u*−1_,then *J* = *J*. Otherwise, *J* = *J*+1. The process of dividing batches is shown as Fig 3 [17].

### Judging number of wagons for batch operation

**Fig 4:**
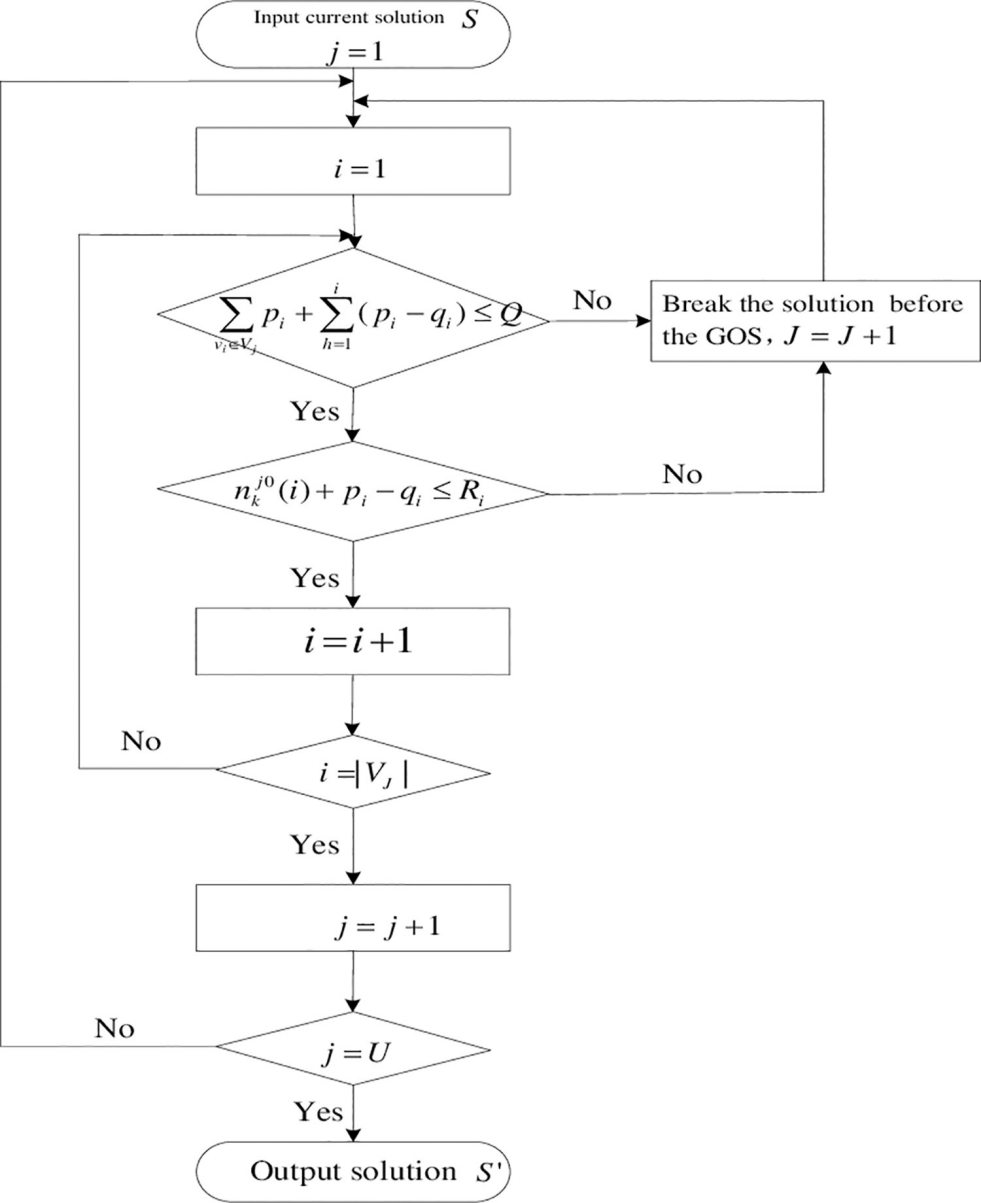
Flow chart of judging number of wagons for batch operation.

Judging number of wagons for batch operation is used to judge whether the traction ability constraint of the shunting locomotive in the current batch is met or not, and whether the capacity of the GOS in the current batch is met or not. Add a batch by breaking the solution *S* before the GOS which exceed capacity of locomotive or GOS, then record the serial number and scale of each batch again. The process of the process of judging number of wagons for the batch operation is shown as Fig 4.

### Computing time of delivering and fetching wagon

The time when the shunting locomotive leaves the station is the start time of the batch operation, and the time when the relevant operations have been finished and the locomotive returns to the station is the end time of the batch operation. The start time of each batch is the key to determine the times of the delivering and fetching of wagons. Compute the allowable start times of each batch according to Constraints (5) ‒ (9), and determine the value of each batch $t_{j}^{start}$ when $y_{i0}(t_{w}-t_{j}^{return})+\sum_{v_{i}\in V_{j}}\max(t_{i}^{finish}-t_{i}^{arrive},0)$ is the minimum. Compute the time $t_{i}^{arrive}$ = $t_{j}^{start}+t_{0i}$ when the locomotive arrives at the first GOS of the batch of operations, and the time when the locomotive arrives at another GOS $t_{i}^{leave}=\max\{t_{i}^{arrive},t_{i}^{finish}\}$. Determine the time $t_{j}^{return}$ when the wagon group of each batch of operations returns to the GOS. If crossing the main track is needed when the wagon group of the batch returns to the station, $t_{j}^{return}=t_{w}$. Otherwise, $t_{j}^{return}=t_{i}^{leave}+t_{i0}$. Compute the duration of the batch *j* of delivering and fetching wagon operations as $t_{j}^{return}-t_{j}^{start}$, then compute the total time of all the batches of delivering and fetching wagon operations $f=\sum_{j=1}^{J}(t_{j}^{return}-t_{j}^{start})$.

### Simulated annealing algorithm

A simulated annealing algorithm is a random search algorithm based on a Monte Carlo iterative solution strategy, which provides an effective approximate solution for problems with NP complexity. Unlike a local search algorithm, the simulated annealing algorithm chooses inferior solutions with large objective values in the neighborhood with a certain probability. Theoretically, it is a global optimal algorithm [18–21]. Judging and processing reverse of delivering and fetching wagons for the initial feasible solution *S*_0_ to ensure it satisfying the visiting sequence relation. The process of dividing batches and judging number of wagons for batch operation could be used to determine the feasibility of the neighborhood solution.

**Fig 5:**
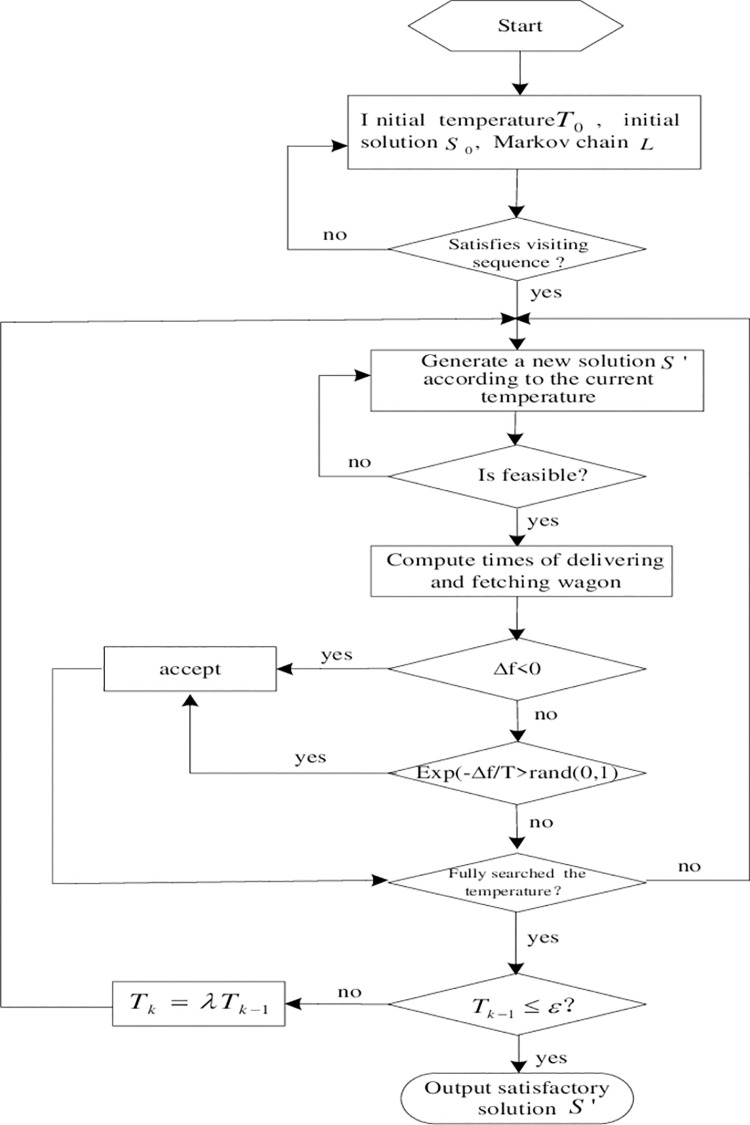
Solution framework of the model.

The computational procedure to the optimization model of delivering and fetching wagons at railway stations with mixed GOSs is shown in Fig 5.The optimization model of delivering and fetching wagons in a railway station with mixed GOSs is a minimization problem, and its objective function *f*(*S*) can be directly used as the evaluation function of solution *S*. In this paper, the neighborhood construction method in the literature [10] is used to construct neighborhood solutions. The new neighborhood solution is constructed by using three methods successively: the forward and backward movement of unloading GOS, the forward and backward movement of loading GOS and 2-exchange.

## Case analysis

### Basic data

**Fig 6:**
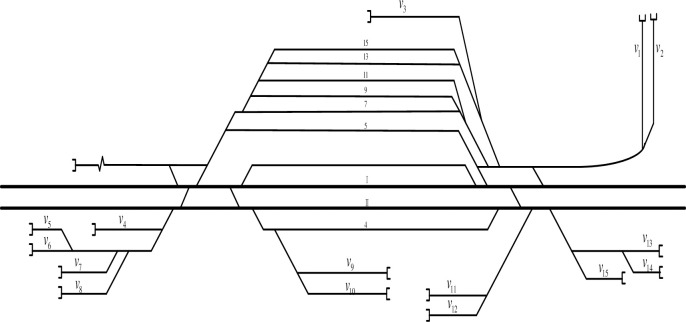
Schematic diagram of the experimental station.

The experimental station is an intermediate station and a freight station, its duties are handling the arrival and departure of regional traffic flow, transshipment trains and some pick-up goods trains, and it is a main center of local city for collecting and distributing goods.The schematic diagram of the experimental station is shown in Fig 6. There are six branches and a total of fifteen GOSs at the experimental station. The shunting yard consists of track 9, track 11, track 13 and track 15.The characteristics of the GOSs are shown in Table 1.

**Table 1:** GOSs of the experimental station.

GOS	Effective length (m)	Capacity *R*_*i*_ (wagons)	Distance to station (m)	Purpose	Branch of GOS	Quadrant	GOS type
*v*_1_	125	8	1730	Unloading	1	I	Special line
*v*_2_	125	8	1730	Loading	1	I	Special line
*v*_3_	213	14	707	Loading	2	I	Goods track
*v*_4_	215	15	942	Unloading	3	III	Goods track
*v*_5_	183	12	2052	Unloading	3	III	Special line
*v*_6_	170	11	2085	Loading	3	III	Special line
*v*_7_	240	16	1925	Loading	3	III	Special line
*v*_8_	145	10	2422	Unloading	3	III	Special line
*v*_9_	182	12	891	Unloading	4	III	Goods track
*v*_10_	190	13	995	Loading	4	III	Goods track
*v*_11_	208	14	1325	Unloading	5	IV	Special line
*v*_12_	189	13	1345	Loading	5	IV	Special line
*v*_13_	230	16	2765	Loading	6	IV	Special line
*v*_14_	258	18	3279	Unloading	6	IV	Special line
*v*_15_	210	14	3355	Unloading	6	IV	Special line

### Experimental scene

We select representative shunting operations of delivering and fetching wagons in the planning period of [6:00 am,10:00 am] on a certain day for testing. Technical characteristics of delivering and fetching wagon operation in the railway station are shown in Table 2. Some tasks of shunting operation for delivering and fetching wagons are shown in Table 3, among which six wagons from GOS *v*_5_ need to be transferred to GOS *v*_3_,and three wagons from GOS *v*_11_ need to be transferred to GOS *v*_10_ for loading goods. The current situation at 6.00is: six wagons at GOS *v*_1_, six wagons at GOS *v*_5_,seven wagons at GOS *v*_6_, eight wagons at GOS *v*_7_, three wagons at *v*_11_, eight wagons at GOS *v*_12_, seven wagons at GOS *v*_14_ and four wagons at GOS *v*_15_. Nine wagons at track 13, among which five wagons should be sent to GOS *v*_2_ and four wagons should be sent to GOS *v*_13_. MATLAB7.0 was used to carry out computer-aided computation on a PC AMD2.20 GHZ computer.

**Table 2:** Technical characteristics of delivering and fetching wagons operation.

Parameters	Symbol	Value
Maximum traction capacity	*Q*	35 wagons
Time for traversing the main track	*t* ^*traverse*^	5 min
Time for selecting wagon group	$t_{t}^{j}$	4 min
Time for splitting wagon group	$t_{f}^{j}$	4 min
Time for collecting wagons	$t_{s}^{j}$	2 min
Time for adjusting position of wagons	$t_{d}^{j}$	1 min
Average running speed of the locomotive	*v*	5×10^2^m /min (30km/h)
Shunting time-window	$\left[t_{w},t_{w}^{'}\right]$	[6:00,6:35], [6:55,7:30], [8:03,8:25],[8:45,9:20]

**Table 3:** Operation tasks of delivering and fetching wagons in the planning period of [6:00 am,10:00 am].

Serial number of operation	GOS	Operation type	Number of wagons	Time of disassembling ($t_{i}^{arr}$)	Finish time of loading or unloading goods ($t_{i}^{finish}$)	Start time of marshalling ($t_{i}^{mar}$)
1	*v*_1_	Fetching	6		6:46	8:49
2	*v*_2_	Delivering	5	6:00		
3	*v*_3_	Delivering	6	7:45		
4	*v*_4_	Delivering	2	6:30		
5	*v*_5_	Fetching	6		7:45	
6	*v*_6_	Fetching	7		8:05	8:49
7	*v*_7_	Fetching	8		7:50	8:49
8	*v*_8_	Delivering	3	7:17		
9	*v*_9_	Delivering	5	7:17		
10	*v*_10_	Delivering	3	8:10		
11	*v*_11_	Fetching	3		8:10	
12	*v*_12_	Fetching	8		7:35	9:35
13	*v*_13_	Delivering	4	6:00		
14	*v*_14_	Fetching	7		7:48	9:16
15	*v*_15_	Fetching	4		8:02	9:16

### Algorithm comparison

We convert the time of day into the corresponding number in [0,1440]. To verify the performance of the algorithm, genetic algorithm(GA) and tabu search algorithm (TS) are used as comparison. Set the parameters of simulated annealing algorithm(SA), genetic algorithm(GA) and tabu search algorithm as Table 4.

**Table 4:** Parameters of the three algorithms.

SA		GA		TS	
Markov chain *L*	50	Group size *NP*	100	Tabu length *TabuL*	20
Initial temperature *T*_0_	1×10^6^	Crossover probability *P*_*c*_	0.5	Number of candidate set *Ca*	50
Termination condition *ε*	1×10^-3^	Mutation probability *P*_*m*_	0.01	Maximum iterations *G*	200
Temperature dropping-rate *λ*	0.99	Generation of termination *G*	200		

The above three algorithms are used as the main framework for programming. The objective function of the model is used as the quality evaluation function of the solution, and process of dividing batches and judging number of wagons for batch operation are used to determine the feasibility of the neighborhood solution. The final results of the three algorithms are shown in the Table 5, and the computing performance of them are compared in the Table 6.

**Table 5:** Satisfactory operation plans obtained from the three algorithms.

Algorithm	Satisfactory solution
SA	$g_{1}^{1}(2)g_{1}^{3}(1)g_{3}^{1}(4)g_{3}^{3}(5)g_{2}^{1}(3)g_{4}^{1}(9)g_{3}^{1}(8)g_{3}^{3}(6)g_{3}^{3}(7)g_{5}^{3}(12g_{5}^{3}(11)g_{4}^{1}(10)g_{6}^{1}(13)g_{6}^{3}(14)g_{6}^{3}(15))$
GA	$g_{1}^{1}(2)g_{1}^{3}(1)g_{3}^{3}(5)g_{3}^{1}(4)g_{2}^{2}(3)g_{4}^{1}(9)g_{6}^{1}(13)g_{3}^{3}(6)g_{3}^{1}(8)g_{3}^{3}(7)g_{5}^{3}(12)g_{5}^{3}(11)g_{4}^{1}(10)g_{6}^{3}(14)g_{6}^{3}(15)$
TS	$g_{1}^{1}(2)g_{1}^{3}(1)g_{3}^{1}(4)g_{6}^{1}(13)g_{3}^{3}(5)g_{3}^{3}(6)g_{2}(3)g_{4}^{1}(9)g_{3}^{1}(8)g_{5}^{3}(11)g_{3}^{3}(7)g_{5}^{3}(12g_{4}^{1}(10)g_{6}^{3}(14)g_{6}^{3}(15))$

**Table 6:** Comparison of results of the three algorithms.

Index	SA	GA	TS
Computing time	8seconds	12seconds	13seconds
Running time	118min	125min	131min
Waiting time	8min	13min	18min
Interference time	0min	0 min	0 min

As can be seen from Table 6, the impact on delivering and fetching wagons of receiving and departing train in the planning period are perfectly avoided by the three algorithms. In terms of computing results and speed, simulated annealing algorithm is relatively better than the other two algorithms, which indicates that simulated annealing algorithm is the algorithm that should be recommended firstly for the problem of delivering and fetching wagons at a railway station with mixed-shaped GOSs. The computing time of the algorithm can meet the requirements of planning shunting operations in railway station.

The tasks, in this case, includes the delivering, the fetching and the transferring operations for wagons at a GOS, and therefore it shows that the case is representative and the model proposed in this paper is universal for different operation forms.

## Conclusion

In this paper, for the problem of the delivering and fetching of wagons in a railway station with mixed GOSs, a general optimization model is established, and a simulated annealing algorithm is used as our algorithm to solve the model. Compared with the previous optimization model of delivering and fetching wagons at a railway station, the applicability of the model is more universal, the main characteristics of which are shown as follows.

1. A station with radial-shaped GOSs and branch-shaped GOSs can be considered as a special form of a station with mixed-shaped GOSs. Therefore the model provided in this paper is also applicable to other layout forms of GOSs.
2. Since delivering wagons separately, fetching wagons separately, delivering and transferring wagons in combination, fetching and transferring wagons in combination, and fetching and delivering wagons in combination are all special forms of fetching, delivering and transferring in combination, the model proposed in this paper is also applicable to all the above operation forms of delivering and fetching wagons.
3. Since all wagons of a train simultaneously arriving and departing is a special case of wagons arriving and departing at different times, the model proposed in this paper is applicable to all such situations in the planning period.
4. The time for which the shunting locomotive is waiting for loading and unloading goods and the time when arriving and departing trains influence shunting operations are included in the model objective. The visiting sequence, the traction capacity, the start time and the finish time for a batch of shunting operations are all considered in the model, and these make the shunting operation plan more feasible.

In order to conduct further in-depth research on the optimization problem of the delivering and fetching of wagons at a railway station with GOSs, the future research direction is to combine this work with the freight train marshalling rules in railway stations, with the aim of providing more convenient marshalling operations when delivering and fetching wagons.

## Supporting information

S1 Table GOSs of the experimental station. (XLSX)

S2 Table Operation tasks of delivering and fetching wagons in the planning period. (XLSX)
